# On Colour, Category Effects, and Alzheimer's Disease: A Critical Review of Studies and Further Longitudinal Evidence

**DOI:** 10.1155/2015/960725

**Published:** 2015-05-17

**Authors:** F. Javier Moreno-Martínez, Inmaculada C. Rodríguez-Rojo

**Affiliations:** ^1^Departamento de Psicología Básica I, Facultad de Psicología, UNED, Calle Juan del Rosal, No. 10, 28040 Madrid, Spain; ^2^Departamento de Psicología Básica II (Procesos Cognitivos), Facultad de Psicología, Universidad Complutense de Madrid, Campus de Somosaguas, Pozuelo de Alarcón, 28223 Madrid, Spain

## Abstract

The role of colour in object recognition is controversial; in this study, a critical review of previous studies, as well as a longitudinal study, was conducted. We examined whether colour benefits the ability of Alzheimer's disease (AD) patients and normal controls (NC) when naming items differing in colour diagnosticity: living things (LT) versus nonliving things (NLT). Eleven AD patients were evaluated twice with a temporal interval of 3 years; 26 NC were tested once. The participants performed a naming task (colour and greyscale photographs); the impact of nuisance variables (NVs) and potential ceiling effects were also controlled. Our results showed that (i) colour slightly favoured processing of items with higher colour diagnosticity (i.e., LT) in both groups; (ii) AD patients used colour information similarly to NC, retaining this ability over time; (iii) NVs played a significant role as naming predictors in all the participants, relegating domain to a minor plane; and (iv) category effects (better processing of NLT) were present in both groups. Finally, although patients underwent semantic longitudinal impairment, this was independent of colour deterioration. This finding provides better support to the view that colour is effective at the visual rather than at the semantic level of object processing.

## 1. Introduction

Confrontation naming tasks have been broadly used to evaluate the cognitive status of neurological patients [1, 2]. Retrieving the name of an object from a pictorial stimulus requires several steps: perceptual analysis of the visual input, access to semantic information on that object, recovery of its lexical label, and, finally, access to its phonological form [3–5]. Thus, an apparent effortless task such as naming an item involves many pivotal cognitive processes. Obviously, inappropriate functioning of one or several stages will significantly impact patients' naming ability.

Models of object recognition and ulterior effective naming can be broadly described as those stressing importance of edge information—as necessary and sufficient to achieve correct identification [6, 7]—and those suggesting an additional role for surface object properties (e.g., colour information [8]). According to the first view, object recognition is* exclusively* based on information about object shape, which, eventually, permits detecting perceptual cues that are central for visual identification. Any other surface attributes, like colour, not only are unnecessary but could even disrupt ordinary recognition of objects [6, 9]. Contrariwise, models giving an additional role to other surface characteristics, such as colour, defend that such supplementary information also contributes to identification of objects, particularly under certain circumstances. For example, according to Tanaka and coworkers, colour confers advantages to recognition when it is a* diagnostic characteristic *of an object, that is, when colour is symptomatic of this object and, additionally, not many objects share this same colour [8, 10]. This group of researchers found that objects with high colour diagnosticity (e.g., a banana) show a stronger effect of colour in recognition when compared to objects with low colour diagnosticity (e.g., a lamp). Thus, colour would confer an advantage for the recognition of living things (LT), such as animals or fruits, with respect to nonliving things (NLT), such as furniture or vehicles. This is because LT usually have higher colour diagnosticity than NLT. Thus, colour is a source of important information for recognising LT; however, for NLT, its role is virtually negligible.

Research on the role of colour as favouring or disrupting object recognition is controversial, both in healthy and in neurological patients (for reviews, see [11, 12]). The seminal study by Bisiach [13] on the effect of colour on aphasic patients found better naming of coloured pictured of objects, with respect to uncoloured line drawings. Several studies with agnostic patients support the idea that colour benefits recognition, especially in objects with high colour diagnosticity: commonly, LT compared to NLT [8]. This view contrasts with a meta-analytic review on naming in Alzheimer's disease (AD) [14]. The authors reported a significant impact of stimulus colour on the effect size for LT; paradoxically, however, the presence of colour* deteriorated* the naming of LT in AD patients; we will turn to this shortly.

Neuropsychological studies on colour in AD are closely related to category effects: the relative loss of cognitive performance in one domain of knowledge (e.g., LT) with respect to another (e.g., NLT). At present, the existence of this phenomenon in AD is strongly debated. Although seminal studies reported category effects in AD [15], current works suggest these effects are rare [14, 16–22]. Thus, in their meta-analytic review, Laws et al. [14] found more reports of LT deficits (i.e., the more commonly reported outcome); however these last ones were in fact no larger—in terms of effect size—than the loss associated with NLT naming. Irrespectively of the rarity or regularity of category effects in AD, it seems logical that these patients benefit from receiving supplementary cues, such as colour, when naming certain items. Thus, colour should assist the naming of items with high colour diagnosticity (LT) in this population.

Certainly, the view that colour affects naming in patients negatively seems counterintuitive, particularly when they name LT items. However, the investigation on this topic is far from unambiguous, with studies reporting patients improving and not improving naming ability with colour items. For example, in a study with a group of AD patients [23], the authors reported impairment in LT relative to NLT, when evaluated with black-and-white line drawings. However, there were no LT/NLT differences either in the controls or in the patients when coloured pictures were utilised. The authors proposed that colour is crucial to process semantic information, because it differentially affects naming of LT and NLT, both in healthy controls and in patients. Chainay and Rosenthal [24] reported on five patients who showed clear LT/NLT naming differences when black-and-white drawings were used; however, they observed that colour stimuli facilitated naming of LT, but not of NLT items. Additionally, another study with 10 AD patients evaluated with line drawings and colour photographs [25] observed a disproportionately worse naming of LT, compared to NLT items, but only when line drawings were presented. Thus, similarly to previous studies, colour facilitated naming of LT, but not—or not to the same degree—that of NLT. In contrast, a further study by Adlington et al. [26] observed the naming ability of 41 AD patients. They were evaluated with exactly the same items presented in three formats: line drawings and greyscale and colour photographs. The authors observed that whilst healthy controls improved with progressive addition of details (i.e., line drawings to greyscale and this to colour photographs), patients did not. The authors interpreted this in the sense that AD patients showed no benefit with the addition of structural cues or even when providing colour information. As mentioned above, a similar result was reported in the meta-analytic study by Laws et al. [14].

Nevertheless, some methodological aspects might have contributed to whether or not studies find benefit from colour in AD patients and, eventually, whether category effects are found. Two of these reasons, closely related to each other, were proposed by Laws and colleagues (see, e.g., [27, 28]). The first one concerns the consequences of failing to use a control group to compare patients' outcomes. Accordingly, using within-patient analysis, without conducting additional comparison with a control group, will distort the presence of LT/NLT deficits. This means that the absolute LT/NLT intragroup difference is a confounding indicator of the presence or even of the direction of category effects [28]. In this regard, Chainay and Rosenthal [24] only presented within-patient analysis and no control data were reported. Thus, it is difficult to know whether the observed LT/NLT differences had enough weight to be considered true category effects (see [27, 28]).

Problems derived from ceiling effects in controls are another concern addressed by Laws and coworkers; this difficulty is mainly derived from using simple line-drawn items [18, 19, 28]. As reported by Laws et al., the presence of ceiling effects in studies with AD patients distorts the number and direction of category effects. Laws et al. [28] observed that items producing ceiling effects overestimated LT impairments; in contrast, these same items undervalued NLT deficits. In this regard, the outcome reported by Montanes et al. [23] and Zannino et al. [25]—that is, presence of worse naming of LT compared to NLT in AD patients—might be questioned because of the presence of ceiling effect in controls. Thus, in the study of Montanes et al., controls named 96% and 97% of LT and NLT items, respectively (Table 1, p. 43). Likewise, in the study of Zannino et al., healthy controls correctly named 91% and 95% of LT and NLT items in colour and 83% and 95% of LT and NLT items in greyscale photographs (data derived from Table 2, p. 1836). Hence, it is presumable that controls reached ceiling when they named all except for the LT/greyscale items. As a result, it is likely that an overestimation of LT impairments was reported in the two mentioned studies [28], mainly when formats that facilitate naming were used (i.e., colour items). Certainly, the fact that normal controls reach the ceiling of the scale (when naming items from any domain) makes it difficult to conduct reliable group comparisons.

Finally, in the study by Adlington et al. [26], the authors concluded that AD patients presented no benefit when provided with colour information. Indeed, main effects concerning domain support this claim: healthy controls increased naming performance when additional cues were presented: colour items (68%) are better than greyscale items (59%) and greyscale items are better than line-drawn items (50%). In contrast, AD patients presented no differences when naming colour (34%), greyscale (33%), or line-drawn (30%) items. It is worth noting that although nonsignificant differences were reported, their pattern was quite similar to that of controls: colour > greyscale > line-drawn. More importantly, however, is the Format × Domain interaction, where both groups presented* similar* patterns. Thus,* all the participants* showed clear LT/NLT differences when naming greyscale and line-drawn items, but not when colour stimuli were presented. This means that whereas NLT were better named than LT with greyscale and line-drawn items, differences between domains disappeared when colour items were utilised. Thus, the conclusion that AD patients cannot benefit from addition of colour should be further detailed in this sense: although AD patients did not* globally* improve with colour addition, they (like normal controls) could benefit when naming items with high colour diagnosticity, that is, those of LT.

Different accounts of category effects have been proposed, and a detailed review of them is not our concern here (for a review, see [29]). However, the view that category effects reflect that identification of some items requires greater cognitive effort (LT versus NLT, resp.) is far from new [30–32]. The crucial point here is that category effects mirror the influence of intrinsic factors or nuisance variables (NVs) which make some categories cognitively more challenging. For example, LT tend to have lower familiarity, word frequency, and higher visual complexity than NLT [30–32]. Likewise, it has been proposed that LT are inherently more similar than NLT; thus, LT would be harder to discriminate (and, hence, to name) compared to NLT [33]. Additionally, LT may present a smaller semantic distance between their members than that of NLT, due to the fact that the former have a higher degree of overlapping among their semantic attributes than the latter; this would make it harder to discriminate LT members compared to NLT members [34, 35].

In this respect, a consistent finding in studies of NVs is that they play a major role in the explanation of the naming performance of AD patients with regard to domain; some works even reported NVs being the only significant predictors. For example, a study by Tippett et al. [22] found that NVs accounted for 25 and 40% of naming variance of NC and AD patients, respectively; alternatively, domain (in their study, category) was not a significant predictor in any group. Gale et al. [16] found that, compared to domain, NVs explained threefold naming variance in AD patients (33% versus 11%). This imbalance was even more noticeable in a longitudinal study by our group: up to 71% for NVs versus about 8% for domain [17].

Consequently, this study was conducted in an attempt to elucidate the dispute about the role of colour in normal and pathological naming, taking into account the aforementioned concerns. We examined the naming evolution of a group of AD patients compared to a group of healthy matched controls. AD patients were evaluated twice with an interval of approximately three years. Participants were presented with the same items with two different formats: colour and greyscale photographs. We attempted to ascertain the role of colour in category effects in AD patients and healthy people, when the influence of potential NVs is also considered, as well as to longitudinally verify the potential influence of such NVs when participants name colour and greyscale items. Likewise, in order to avoid difficulties derived from ceiling effects in controls which could “overshadow” the results, a set of LT/NLT items selected to deal with this problem and matched across domains in several NVs was used.

## 2. Methods 

### 2.1. Participants

Thirty-seven Spanish-speaking participants took part in this study: 11 patients (10 females, 1 male) with probable AD (AD-1 and AD-2, according to the moment of evaluation) and 26 healthy normal controls (NC: 13 females, 13 males). As described in Procedure, NC participants were assigned to one of two naming conditions: colour and greyscale. The patient groups did not differ statistically from the control participants with regard to age *F*_(3,47)_ = 1.9, *P* = .1 or educational level *F*_(3,47)_ = 2.2, *P* = .1. The Mini Mental State Examination (MMSE [36]) scores, after correcting for age and educational level in the Spanish population [37], were significantly lower in the AD group than in NC (*F*_(3,47)_ = 21.7, *P* < .0001), although MMSE scores did not differ between the two groups of patients.  Table 1 shows the demographics characteristics of the participants.

All patients were diagnosed by Spanish senior neurologists after undergoing neurological examination, laboratory tests, and brain imaging to rule out other possible causes of dementia. All patients fulfilled NINCDS-ADRDA [38] and DSM-IV-TR [39] criteria for probable AD. No patient presented depression or any other medical or neurological condition known to impact cognitive performance. The NC group consisted of healthy elderly volunteers with no history of alcoholism, drug abuse, and psychiatric or neurological disorders. All the participants had normal or corrected-to-normal vision. Prior to data collection, the study protocol was approved by local institutional review boards; thus, any human data included in this paper was obtained in compliance with regulations of our institution and it conforms to the Declaration of Helsinki. All participants or their families (in cases of diminished capacity) gave their informed consent to participate in the study. An additional exclusion criterion for NC was a MMSE score below 25, in order to discard participants with potential cognitive impairment [37]. Finally, any potential participant presenting problems to accurately perceive colours was excluded from the study.

### 2.2. Procedure

To avoid potential priming effects across conditions—which could occur if NC had seen the two sets of photographs consecutively—NC were pseudorandomly assigned to one of two naming conditions (i.e., colour—7 females and 6 men—and greyscale—6 females and 7 men), which varied according to the image format used in the naming task. In the colour condition, participants were presented with colour versions; in the greyscale condition, they were presented with greyscale versions of the same items. The AD patients were requested to complete both naming tasks (i.e., the colour and greyscale versions). Half of the AD patients in each group (i.e., AD-1, AD-2) named the colour photograph first followed by the greyscale version, and half of the patients performed the two tasks in the reverse order. For each AD patient, there was a minimum 2-week delay (maximum 4 weeks) between the two testing sessions (i.e., colour and greyscale versions). As mentioned, the patients were evaluated twice with a temporal interval of approximately three years; the NC participants were evaluated once.

**Figure 1:**
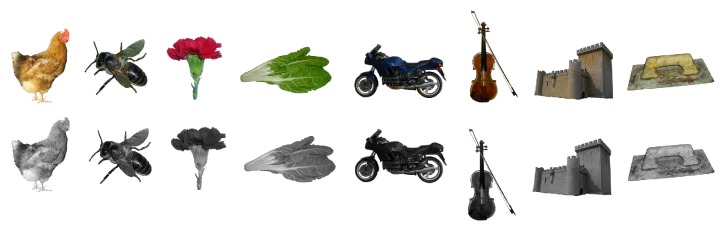
Colour and greyscale versions of items from the Nombela Naming Test. From left to right: hen, bee, carnation, chard, motorbike, violin, castle, and trowel.

One of the conditions comprised 98 colour photographs selected from the* Nombela Naming test* [40]: 49 LT (7 animals, 7 body parts, 7 flowers, 7 insects, 7 fruits, 7 trees, and 7 vegetables items) and 49 NLT (7 buildings, 7 clothing, 7 furniture, 7 kitchen utensils, 7 musical instruments, 7 tools, and 7 vehicles items). For the second condition, greyscale versions of the same items were used.  Figure 1 shows examples of images presented in colour and greyscale formats; a list of the items is included in the Appendix. Items were matched across LT and NLT domains for age of acquisition (AoA), familiarity, lexical frequency, name agreement, typicality, and visual complexity (Table 2).

The images were presented randomly one by one on a computer monitor, where they remained until the participant gave a response; this was followed by a three-second interstimulus interval before the next item appeared. If no answer was forthcoming, there was a 10-second interval before moving on to the next item. The mean dimensions of each image were 265 × 223 pixels. The experimenter sat beside the participants, recorded their response on the computer keyboard, and pressed a key to begin the next trial. Responses were considered correct when the participant gave the name of the stimulus or other names considered (by the authors and an independent judge) to be synonymous with the target item name, for example, saying “pot” for the stimulus “pan” (in Spanish, cazuela for cacerola). Prior to beginning the 98-item presentation, 6 different practice items were administered to the participants (beetle, ear, helicopter, sail, stool, and tie) to become familiar with the procedure.

## 3. Results

Firstly, skewness and kurtosis statistics (*g*_1_ and *g*_2_) were computed for the NC group (LT and NLT domains) and the two formats. We also calculated the D'Agostino-Pearson omnibus test for normality, which uses both *g*_1_ and *g*_2_ as input. These analyses revealed that the distributions did not differ significantly from normality (Table 3).

To analyze the data, a repeated measures ANOVA was performed with Group (NC/AD-1/AD-2) and Format (colour/greyscale) as within-item factors and Domain (LT/NLT) as between-item factor [25]. A main effect of Group was observed (*F*_(2,96)_ = 92.2, *P* < .0001); post hoc analysis with Bonferroni correction revealed that the average performance of the NC group was higher than that of the two AD groups (NC: *M* = 51%; AD-1: *M* = 33%; AD-2: *M* = 30%). Furthermore, the average performance of the AD-1 group was higher than that of AD-2 group. The effect of Format was significant (*F*_(1,96)_ = 4.3, *P* = .04); post hoc analysis indicated that coloured photographs were better named than greyscale ones (*M* = 39% versus *M* = 37%). The effect of Domain was marginally significant (*F*_(1,96)_ = 3.6, *P* = .06); post hoc analysis showed a tendency to better naming of the NLT with respect to the LT (*M* = 44% versus *M* = 32%, for NLT and LT, resp.).

As regards the outcome of the interactions, the Domain × Format was the only significant one (*F*_(1,96)_ = 5.8, *P* = .02); post hoc analysis indicated that LT were better named when colour photographs were used than when participants named greyscale ones (*M* = 34% versus *M* = 30%, for colour and greyscale, resp.); however, with NLT items, there was no naming improvement with colour compared to greyscale photographs (*M* = 44% versus *M* = 44%, for colour and greyscale, resp.). No other significant interactions were observed: Group × Domain, Group × Format, or Group × Domain × Format (*F* > 1, *P* > .5, in all the conditions).

Analyses show that, similarly to NC, AD patients are also able to take advantage of colour. Clearly, if AD and NC would differ at this point, correlations between colour and greyscale items should clearly differ between groups. Thus, if AD patients would not benefit of colour (or did to a lesser extent than NC), groups of patients and NC would distinguish each other with regard to their pattern of correlations; furthermore, differences should be particularly evident comparing LT and NLT, since colour is a characteristic that supposedly benefited processing of LT. Consequently, correlations between colour and greyscale items were calculated. In NC, correlations between colour and greyscale items were highly significant (*r* = .94, *P* < .0001), as they were for both groups of patients: AD-1: *r* = .93, *P* < .0001, and AD-2: *r* = .93, *P* < .0001. The same tendency was observed when correlations were separately calculated for LT (NC: *r* = .93, *P* < .0001; AD-1: *r* = .90, *P* < .0001; AD-2: *r* = .95, *P* < .0001) and NLT (NC: *r* = .95, *P* < .0001; AD-1: *r* = .95, *P* < .0001; AD-2: *r* = .92, *P* < .0001). Thus, correlations analyses confirm previous results: both NC and AD patients seem to take advantage of colour information when naming items.

Finally, we submitted the naming performance of the AD patients (two time-points) and the NC groups to separate stepwise multiple regression analyses, with the goal of determining the influence of domain and NVs as predictors of naming ability within each format examined: colour and greyscale [20]. Overall accuracy on the single items of the naming task was the dependent variable; the domain (LT/NLT) was coded as a dummy variable; finally, the values of the NVs of the items were the independent variables. Results from regression analyses were in line with those from the previous ANOVA.  Table 4 shows that, usually, the impact of NVs was higher than that of domain. NC better named items with a greater AoA, name agreement, and typicality and those with lower visual complexity. AD patients better named items with higher name agreement, familiarity, earlier AoA, and lower visual complexity. Domain, the second relevant predictor, had a comparatively minor impact on naming, with NLT being more accurately named than LT. Notably, the influence of domain was comparable for AD patients and NC, regardless of the format of the item; thus, NLT items were named more efficiently than LT items. Furthermore, as shown in Table 4, domain exerted similar influence* within* each format, although its impact slightly decreased* between* formats, that is, from greyscale to colour items. This indicates that colour barely beneficiated naming of LT items in* all* the groups.

Results concerning domain are of particular interest because they suggest the following: (1) as a rule, domain is not as strong a naming predictor as are NVs; this is true for patients and NC; (2) despite the existence of quantitative differences among groups, they show the same qualitative pattern: better naming of NLT items, irrespective of the format used; (3) the addition of colour, which should “equilibrate” any potential imbalance between domains, did not do so: differences favouring the naming of NLT items persisted; (4) moreover, the—putative—influence of colour was similar for all the groups, slightly favouring the naming of LT items.

### 3.1. Individual Analysis: Exploring Potential Sex Skewing

As the group of AD patients consists mostly of females (10 : 1), our results may be influenced by the fact that female AD patients present the reported “female advantage” for LT; obviously, this could bias the observations [41–44]. Inspection of raw LT/NLT differences (Table 5) showed that only one female patient (AD-1) displayed no LT/NLT differences when evaluated with colour photographs at time-point-1; the same patient showed an LT advantage when evaluated with greyscale items at time-point-2. This outcome contrasts with the rest of the AD patients who, as a rule, showed an NLT advantage regardless of the format (colour or greyscale) throughout both time-points. In order to further explore this, we examined individual performance to check whether s/he had a significant deficit on one or the other semantic domain. Thus, individual AD patients' performance on LT and NLT was compared to that of the NC. The modified *t*-test by Crawford and Howell [45] revealed no cases of LT/NLT dissociations when AD patients were evaluated with greyscale and colour photographs.

## 4. Discussion

In this study, we observed the ability of AD patients and NC to use colour information when naming items from LT and NLT. In addition, the impact of NVs on naming, as well as potential problems derived from ceiling effects in NC, which may have influenced previous studies on this topic, was also considered. Eleven patients were evaluated twice with an interval of about three years. The participants were assessed with exactly the same items presented in two formats: colour and greyscale. In addition, the items were matched across domain on the following NVs: AoA, familiarity, lexical frequency, name agreement, prototypicality, and visual complexity. Results indicate the following: (i) relationship between colour and semantic domain: colour slightly favours processing of items with higher colour diagnosticity, that is, that of LT; (ii) colour similarly helps NC and AD patients; in addition, patients retain this ability over time; (iii) category effects were observed in both groups of participants; these findings are discussed below.

### 4.1. Effects of Colour in AD and Healthy Naming

Studies on the role of colour in object recognition in AD are controversial; on the one hand, works showing that colour deteriorates the naming of LT have been reported [14], as well as studies stating that patients did not benefit from addition of colour [26]. On the other hand, it has been observed that AD patients, like NC, improve their naming when evaluated with colour instead of greyscale or black-and-white items [23–25]. As aforementioned, some of these discrepancies could have a methodological basis. In keeping with previous works, in our study, AD patients and NC both benefit from colour [23–25]. The regression analyses illustrate that the influence of domain mildly decreases in all the participants when they named colour with respect to greyscale items. Thus, the semipartial correlation coefficient decreased in all the groups: NC from .19 to .15; AD-1 from .25 to .17; and AD-3 from .30 to .23. This means that* all* of our participants slightly improved the naming of LT coloured items with respect to greyscale ones. However, differentially to studies reporting the disappearance of category effects when AD patients named LT colour items [23–25], in our study, category effects persisted in all the groups; in other words, the improvement derived from adding colour was insufficient to balance the LT/NLT disparity. Consequently, as the absence of ceiling effects is the most significant dissimilarity between our study and the above-mentioned studies, we postulate that this could be the essential point: it is possible that NC would have shown the same LT/NLT disparity with no ceiling effects present. This would be in keeping with studies stressing that ceiling effects make it difficult to conduct reliable comparisons between groups, distorting the presence or direction of category effects [27, 28].

It has been suggested that the use of greyscale instead of colour items could somehow amplify preexistent LT/NLT differences [25]. LT items would require a higher degree of cognitive processing than NLT items, because they are intrinsically more similar [33, 35] or cognitively more challenging [30–32]. As a result, a controversy about which is the* normal profile* in neurologically intact individuals is patent in this arena. Accordingly, the domain that should be normally better processed by healthy controls is discussed. On the one hand, better and faster naming of LT than NLT has been reported [46–53]. On the other hand, studies showing an advantage for naming NLT have also been reported, defending a better “normal” processing of NLT [54, 55]. In our study, LT items were, in fact, more difficult to process than NLT items; this was true regardless of the format of the item and, more importantly, the cognitive status of the participant. Additionally, stability concerning the influence of domain persisted in patients, as the imbalance between domains favouring NLT persisted over time. Therefore, our results provide arguments concerning the controversy about the “normal profile” and lend support to a better normal processing of NLT instead of LT items in naming tasks.

The presence of low level visual damage in AD has been proposed as a plausible explanation for the LT/NLT naming imbalance when different formats of items are used [26]. Consequently, AD patients would not benefit from colour because of vision problems associated with this pathology. Indeed, pathological changes affecting the eye, optic nerve, and visual cortex have been reported in AD [56], as well as impairment in perception of colour and further visual difficulties [57]. Certainly, our results do not support this view. Indeed, according to the above approach, the occurrence of deficits in colour vision, or further visual difficulties in patients, should have undermined, instead of helping, the naming of items with high colour diagnosticity. Noticeably, this was not the case. Clearly, the vision impairment view is not adequate to explain the fact that NC showed the same qualitative profile in LT items as AD patients. It should be acknowledged that although colour vision was not objectively assessed with a test of colour vision—but through a personal/subjective statement—the fact that colour similarly affected performance of patients and controls supports the lack of deficit in colour vision in the group of patients.

In this context, there is controversy about whether visual deterioration has an impact on cognitive functions in AD. Thus, there have been reports that colour and stereoacuity deficits are unrelated to severity of dementia [58]; visual deficits in AD affect specific cognitive domains [59]; or semantic and naming problems coexist with normal visuospatial perception [60]. In this regard, our data show that progressive decline in naming can coexist with stable MMSE scores and with—presumed—lack of colour vision problems [58]. Additionally, our study suggests that a general measure of dementia severity (i.e., MMSE) may overlook slight cognitive decline, such as that objectified through the picture naming task [61]. This would agree with one recent study by our group showing that whereas MMSE was unable to differentiate NC from MCI patients, the picture naming task successfully performed such subtle discriminations [62].

Controversy on whether colour is effective either at the visual or at the semantic level of object processing is present in the literature. Some proposals defend that information about colour could be located at the level of structural representations of objects, while semantic information refers exclusively to functional and associative knowledge about an object [3]. Accordingly, knowing whether a strawberry is red or blue would require accessing the structural long-term storage, whereas knowing whether a strawberry can be used to make marmalade would imply accessing semantic information [3]. Other views suggest that colour information could be doubly stored, as verbal-semantic and visuosemantic information [63, 64], or that the effect of colour and additional visual cues in naming would be related to the putative localization of the perceptual information: visual or semantic [65]. Accordingly, information about colour would play a role at the semantic level, while photographic details would exert their action through the structural description level [65]. Our data do not support this last view; we reason that if AD patients suffer from semantic deficit, the derived anomie should be putatively semantic in nature [1, 66]. Consequently, if colour acted at the semantic level, information about colour should be associated with semantic impairment. Therefore, semantic erosion would be associated with colour deterioration, and the longitudinal decline of semantic information observed in our study should have been associated with deterioration of colour. In contrast, if the information about colour is located in the structural description storage, semantic impairment and colour knowledge could dissociate from each other; thus, the former could present deterioration but not necessarily the latter. As mentioned, our AD patients underwent longitudinal semantic impairment, which was putatively independent of colour deterioration. Furthermore, patients' qualitative performance showed temporal stability, as it did not differ substantially over time; thus, the potential influence of colour did not vary appreciably over time. This suggests that the influence of colour was mainly exerted through the structural level [3].

Inferences deriving from the variable visual complexity further support the above-mentioned conclusion. Visual complexity reflects the amount of detail, intricacy of lines, pattern, and quantity of colours (in the case of coloured images) presented in a particular image [67]. Stewart et al. [32] first demonstrated that picture naming is modulated by visual complexity of the items, influencing the fact of whether or not category effects are found in healthy participants. Clearly, according to a model of naming that involves different stages (i.e., perceptual, semantic, lexical, and phonological [3]), visual complexity will primarily affect the perceptual level [68]. Consequently, if one assumes that the anomie presented by our patients derived* mostly* from perceptual disturbances (instead of being semantic in nature), a predominant role of visual complexity, with respect to the other NVs, should have been found in patients. As shown in Table 4, visual complexity exerted some influence on participants' performance (both NC and patients), but it was neither the only nor the main NVs affecting performance; additionally, the influence of visual complexity was similar between NC and patients. Finally, if photographic details exert their action through the structural description level [65], the influence of visual complexity should have been particularly patent on greyscale as compared to colour items; accordingly, its influence should have become differentially apparent on greyscale compared to that on colour items. As shown in Table 4, our data do not seem to support this view: no clear major differences regarding visual complexity were detected between the two formats. Thus, additional evidence coming from visual complexity further supports the aforementioned conclusion: in our study, the influence of colour was mainly exerted through the structural—instead of the semantic—level. Certainly, we do not intend to have the final say on this topic because our goal did not focus on this controversial matter; however, our results provide better support to the view that colour is effective at the visual level of object processing [3] rather than at the semantic stage [65].

### 4.2. Naming and Category Effects in Participants

This study expands upon previous works on AD by showing that this pathology is associated with an evident damage to the naming ability, which is supposedly semantic in nature [1, 66, 69–71]. Additionally, naming difficulties of our patients increase with the severity of dementia, which is in line with previous longitudinal works on this topic [17, 72–74]. Furthermore, the naming ability of AD patients is lower than that of NC, irrespective of the format used to evaluate them, that is, regardless of whether colour, greyscale images—or black-and-white line drawings—are used [25, 26].

Despite the fact that there is clear agreement that AD erodes semantic information, the literature on the presence of category-specific effects is still conflicting. Most of the studies have reported LT deficits, a minority have reported NLT deficits, some report both, and still others find no category-specific effects in AD (for a review, see [75]). A recent meta-analytic review found that the belief of a greater incidence of LT deficits in AD patients may be misleading [14]. These authors found more studies reporting LT deficits (i.e., the typically more reported outcome); however, no significant difference in the effect sizes for naming LT and NLT was observed. Additionally, naming studies showing a similar patients-to-control ratio of category effects have been reported [21]; similarly, the infrequency of category effects both in NC and in AD patients has been advocated [17]. Our results, showing the presence of category effects in all the participants, provide support for these notions. In addition, the fact that patients and NC both presented category effects lends support to the view that LT/NLT differences between both populations are only quantitative but not qualitative in essence [14, 16–21].

### 4.3. The Role of NVs in Naming Picture

The literature on category effects reflects disparity about the number of cases presenting LT/NLT impairments; thus, the quantity of LT deficits has been estimated as five times higher than that of NLT [28]. It must be considered that several works on this topic did not control important NVs (see [76]); evidently, some of these variables were unknown for the first studies in this field. Consequently, some caution should be taken when considering the putative LT/NLT asymmetry, especially when relevant NVs, such as familiarity or visual complexity, were not considered [76]. Our results support this view: despite the fact that domain was a significant predictor for all the groups, its impact was comparatively inferior to that of NVs. In this regard, recent studies with AD patients have claimed that NVs are better naming predictors than domain [16, 17]; some researchers even reported NVs to be the only significant predictors regardless of domain [22]. Apart from supporting this view, our study extends this conclusion from AD patients to healthy participants.

### 4.4. Limitations of the Study

To conclude, two potential limitations in the current study should be commented. The first one concerns the number of patients studied, which while acceptable from a neuropsychological view should invite one to be cautious when generalizing the results. A larger group of patients would likely have permitted extraction of (more) consistent findings, allowing for further evaluations to be conducted. Unfortunately, we are restricted by the inherent nature of longitudinal studies and the occurrence of undesired effects, such as attrition and experimental mortality, which allowed no additional evaluations on this sample.

A priori, the gender of—most of—the patients might be considered another potential limitation. As it is known that females—both neurologically intact individuals and patients—may show advantages when processing LT items, our results might be biased in this respect [41–44]. Given that individual analyses revealed no patient presenting category-specific effects at any temporal point or with any format of presentation, it is likely this “unpleasant” influence of gender can be ruled out from our study. In any case, it is worth noting that, in the worst-case scenario, any potential influence of gender should have positively affected LT, contributing to equilibrate LT/NLT differences, which was not the case.

In summary, our study shows that AD patients use colour information similarly to NC and patients retain this ability over time. In addition, NVs play a significant role as naming predictors in all the participants, relegating domain to a secondary plane. Additionally, despite the fact that category effects were found in AD patients, these were also found in the group of neurologically intact participants; therefore, both patterns were qualitatively comparable. Finally, concerning the debate about whether colour is effective at the visual or at the semantic level of object processing, our study lends support to the former rather than to the latter view.

## Figures and Tables

**Table 1:** Background information about the participants in the study (means and standard deviation, in brackets).

	NC-C	NC-G	AD-1	AD-2
Gender (m/f)	7/6	6/7	1/10	∗
Years of education	4.8 (0.9)	5.4 (2.6)	5.8 (0.4)	∗
Age	77.5 (6.4)	74.5 (3.7)	75.8 (4.0)	78.7 (4.3)
MMSE	28.5 (1.5)	29.5 (1.3)	21.3 (4.8)	19.2 (6.0)

*Note*. NC-C = normal control group (colour version), NC-G = normal control group (greyscale version), AD-1/AD-2 = Alzheimer's disease group (first and second time-points), and MMSE = Mini Mental State Examination.

^∗^Same values as AD-1.

**Table 2:** Matching variables for LT and NLT stimuli (means and standard deviation, in brackets).

	LT	NLT	*P*
AoA	4.2 (1.3)	4.5 (1.5)	.5
Fam.	3.1 (0.9)	3.1 (1.1)	.9
LF	14.7 (1.2)	14.7 (2.5)	.9
NA	0.5 (0.3)	0.6 (0.3)	.6
Prot.	3.5 (0.9)	3.3 (1.1)	.4
VC	2.7 (0.7)	2.9 (0.8)	.2

*Note*. AoA = age of acquisition, fam. = familiarity, LF = lexical frequency, NA = name agreement, prot. = prototypicality, VC = visual complexity, LT = living things, and NLT = nonliving things.

**Table 3:** Distribution statistics for normal controls for greyscale and colour items and the two domains: living things (LT) and nonliving things (NLT).

	Greyscale		Colour	
	LT	NLT	LT	NLT
Skewness *g*_1_	0.17	−0.60	0.18	−0.41
Kurtosis *g*_2_	1.79	1.87	1.80	1.90
D'Agostino-Pearson omnibus test *K* ^2^	3.25	3.86	3.27	3.77
*P*	.20	.14	.19	.15

**Table 4:** Independent predictors of naming performance, greyscale and colour items, in stepwise multiple regressions for controls (NC) and AD patients (two time-points); *r*_*s*_ = semipartial correlation coefficient.

Greyscale items						
	Controls		AD-1		AD-2	
*R* ^2^ adjusted	.59		.46		.50	
(*P*)	*F*_(7,90)_ 35.8 (.0001)		*F*_(7,90)_ 42.7 (.0001)		*F*_(7,90)_ 33.5 (.0001)	

	*r*_*s*_	*P*	*r*_*s*_	*P*	*r*_*s*_	*P*

Age of acquisition	−.14	.18	−.19	.07	−.60	.0001^∗^
Familiarity	.02	.80	.64	.0001^∗^	.01	.35
Lexical frequency	.09	.40	.06	.6	.16	.12
Name agreement	.24	.0001^∗^	.15	.13	.08	.40
Typicality	.27	.0001^∗^	.06	.53	.01	.90
Visual complexity	−.14	.03^∗^	−.14	.17	−.20	.007^∗^
Domain	.19	.04^∗^	.25	.001^∗^	.30	.0001^∗^

Colour items						
	Controls		AD-1		AD-2	

*R* ^2^ adjusted	.58		.53		.53	
(*P*)	*F*_(7,90)_ 34.4 (.0001)		*F*_(7,90)_ 27.9 (.0001)		*F*_(7,90)_ 28.9 (.0001)	

	*r*_*s*_	*P*	*r*_*s*_	*P*	*r*_*s*_	*P*

Age of acquisition	−.28	.0001^∗^	−.01	.35	−.19	.007^∗^
Familiarity	.13	.22	.26	.0001^∗^	.12	.08
Lexical frequency	.01	.35	.15	.15	.04	.70
Name agreement	.13	.04^∗^	.19	.009^∗^	.01	.34
Typicality	.13	.20	−.01	.90	−.04	.73
Visual complexity	−.22	.001^∗^	−.14	.04^∗^	−.14	.04^∗^
Domain	.15	.02^∗^	.17	.01^∗^	.23	.001^∗^

^∗^Significant effects.

**Table 5:** Individual mean naming performance (percentage of correct responses) of AD patients according to domain (LT/NLT), format (colour/greyscale), and moment of evaluation (time-point-1/2).

	Time-point-1 greyscale (%)			Time-point-1 colour (%)			Time-point-2 greyscale (%)			Time-point-2 colour (%)		
	LT	NLT	LT-NLT	LT	NLT	LT-NLT	LT	NLT	LT-NLT	LT	NLT	LT-NLT
Patient												
AD-1 f	49	53	−4	47	47	0	43	39	4	41	47	−6
AD-2 f	37	45	−8	33	43	−10	31	43	−12	31	43	−12
AD-3 f	33	57	−14	31	51	−20	35	53	−18	18	37	−18
AD-4 m	12	31	−19	18	37	−18	6	20	−14	14	31	−16
AD-3 f	35	47	−12	41	49	−8	24	33	−8	22	31	−8
AD-6 f	18	35	−17	33	43	−10	24	45	−20	27	35	−8
AD-7 f	20	45	−25	35	47	−12	18	41	−22	35	49	−14
AD-8 f	20	39	−19	22	47	−24	20	47	−27	24	41	−16
AD-9 f	18	39	−21	12	22	−10	ne	ne	ne	ne	ne	ne
AD-10 f	10	22	−12	22	27	−4	10	22	−12	14	22	−8
AD-11 f	14	33	−19	22	31	−8	6	29	−22	12	24	−12

*Note*. AD = Alzheimer's disease, LT = living things, NLT = nonliving things. LT-NLT = difference between LT and NLT (negative values indicate better naming of NLT), f = female, m = male, and ne = not evaluated.

**Table 6:** Items from the Nombela Naming Test. Within brackets are the original Spanish names.

Living	Nonliving
**Animals**	**Buildings**
Genet (jineta) Hen (gallina) Kangaroo (canguro) Kiwi (kiwi) Ray (raya) Rhinoceros (rinoceronte) Tapir (tapir)	Castle (castillo) Granary (hórreo) House (casa) Pagoda (pagoda) Palace (palacio) Shanty (chabola) Skyscraper (rascacielos)

**Body parts**	**Clothing**
Cerebellum (cerebelo) Kidney (riñón) Liver (hígado) Lung (pulmón) Pelvis (pelvis) Skull (cráneo) Vertebra (vértebra)	Bowler hat (bombín) Coat (abrigo) Girdle (corsé) Kimono (quimono) Panties (pololos) Mitten (mitones) Skirt (falda)

**Flowers**	**Furniture**
Bellflowers (campanillas) Calla lily (cala) Carnation (clavel) Orchid (orquídea) Pansy (pensamiento) Poppy (amapola) Tulip (tulipán)	Night table (mesilla) Bookcase (librería) Couch (diván) Bureau (cómoda) Filing cabinet (archivador) Magazine rack (revistero) Sideboard (aparador)

**Fruits**	**Kitchen utensils**
Medlar (níspero) Melon (melón) Peach (melocotón) Quince (membrillo) Redcurrant (grosellas) Strawberry (fresa) Watermelon (sandía)	Cooking pot (puchero) Chinese colander (Chino) Churrera^∗^ Frying pan (sartén) Peeler (pelador) Pot (olla) Small saucepan (cazo)

**Insects**	**Musical instruments**
Ant (hormiga) Bee (abeja) Butterfly (mariposa) Cockroach (cucaracha) Mosquito (mosquito) Spider (araña) Wasp (avispa)	Clarinet (clarinete) Clavichord (clavicordio) Harp (arpa) Saxophone (saxofón) Trumpet (trompeta) Tuba (tuba) Violin (violín)

**Trees**	**Tools**
Black poplar (chopo) Cypress (cipres) Fir (abeto) Holm oak (encina) Olive tree (olivo) Palm tree (palmera) Willow (sauce)	Cold chisel (cortafríos) Handsaw (serrucho) Pickaxe (alcotana) Pincers (tenazas) Pliers (alicates) Screwdriver (destornillador) Trowel (llana)

**Vegetables**	**Vehicles**
Artichoke (alcachofa) Cabbage (repollo) Celery (apio) Endive (escarola) Leek (puerro) Spinach (espinacas) Cauliflower (coliflor)	Airplane (avión) Bus (autobús) Car (coche) Glider (planeador) Motorbike (motocicleta) Paragliding (parapente) Train (tren)

^∗^Churrera: non-English translation: tool used for making *churros* (fried noodles).

## Appendix

See Table 6.
